# The effect of virtual reality on exercise execution in people with Parkinson’s disease

**DOI:** 10.3389/fpsyg.2026.1809785

**Published:** 2026-05-07

**Authors:** Jana Seuthe, Jule Grotherr, Anna Kauter, Fabio A. Barbieri, Björn Hauptmann, Christian Schlenstedt

**Affiliations:** 1 Institute for Interdisciplinary Exercise Science and Sports Medicine, MSH Medical School Hamburg, Hamburg, Germany; 2 Parkinson’s Disease and Movement Disorders Unit, Department of Neurology, Segeberger Kliniken, Bad Segeberg, Germany; 3 Human Movement Research Laboratory (MOVI-LAB), School of Sciences, Department of Physical Education, São Paulo State University (UNESP), Bauru, São Paulo, Brazil

**Keywords:** balance, Parkinson’s disease, range of motion, rehabilitation, virtual reality

## Abstract

**Introduction:**

Virtual reality (VR) has become increasingly popular for rehabilitation in people with Parkinson’s disease (PD). Despite studies already showing positive effects of prolonged VR interventions, there have been little investigations on the effects of VR on the movement execution, which might impact training intensity. The aim of this study was to investigate the effect of VR on movement execution compared to therapist instructed (TI) exercises in people with PD.

**Methods:**

Thirty people with PD performed three different exercises aimed at improving range of motion (ROM) or postural control in an immersive VR setting. Comparable TI exercises were conducted. ROM and center of pressure were analyzed with a marker-based motion capture system and a pressure plate, respectively, to quantify movement execution. Statistical analysis of was performed using one-way Anova with Bonferroni correction for multiple comparisons and Cohen’s d was calculated as effect size.

**Results:**

For the two exercises to increase ROM results were inconsistent as one exercise showed larger ROM for the VR condition (*p* = 0.009, d = 0.895), whereas the other showed larger ROM during the TI condition (*p* < 0.001, d = 1.955). For the weight-shifting exercise we showed larger and faster displacements of the center of pressure for the TI exercise (*p* < 0.05, d > 0.5).

**Discussion:**

This study demonstrates that the effects of VR on exercise execution differ depending on the specific exercise goal and task characteristics. While fully immersive VR was associated with greater ROM in exercises that benefit from continuous visual feedback, conventional TI exercises resulted in superior performance for other tasks, including balance exercises targeting limits of stability. The findings suggest that visual feedback, perceived safety, and task-specific demands play a crucial role in determining the effectiveness of VR-based interventions. Future research should focus on optimizing VR interventions through adaptive feedback and personalized task difficulty to fully exploit their potential in therapeutic exercise settings.

## 1 Introduction

Virtual reality (VR) has been increasingly investigated and applied in rehabilitation of people with Parkinson’s disease (PD). Among the types of VR, non-immersive, semi-immersive and fully-immersive, the last one is the most capable of constructing a real interactive and fully engaged virtual environment, representing better the real world (Hsu et al., 2022). Studies have already shown that fully-immersive VR interventions can successfully improve balance (Chuang et al., 2022; Kwon et al., 2023; Wang et al., 2021; Canning et al., 2020), gait (Chuang et al., 2022; Dockx et al., 2016; Canning et al., 2020) and motor function (Kashif et al., 2022) of people with PD. Indeed, fully-immersive VR interventions were found to be similarly (Canning et al., 2020) or even more effective (Chuang et al., 2022; Kashif et al., 2022; Wang et al., 2021) compared to conventional rehabilitation. However, fully-immersive VR environments may challenge cognitive-motor integration and little is known how fully-immersive VR interventions affect movement execution.

There are many advantages regarding the use of fully-immersive VR applications for people with PD including the multisensory components of the training, the opportunities to standardize and the potential to design motor-cognitive challenges according to patients’ needs (Canning et al., 2020). In general, VR has the potential to create a motivating environment for training and also help engage patients more (Dockx et al., 2016). In addition, VR applications can implement feedback (Dockx et al., 2016) and can be used to manipulate sensory input which can be beneficial for training (Canning et al., 2020).

However, there may also be disadvantages when VR is used for people with PD. VR environments challenge cognitive-motor integration (Pimenta Silva et al., 2025). In PD, motor automaticity is impaired and individuals with PD often rely on prefrontal areas to compensate for impaired motor automaticity (Li et al., 2025). Reduced cognitive resources may cause worse movement execution when using VR which may impact effectiveness of the treatment. In addition, safety aspects may impact movement execution as fully-immersive VR intervention often requires therapist monitoring for the intervention to be successful, which allows use mostly in a facility-based context (Canning et al., 2020). Especially for the future use in the home environment without therapists monitoring performance, it is necessary to understand how fully-immersive VR interventions impact movement execution compared to therapist-guided performance.

Only few studies have looked into the direct comparison of therapist- versus VR-guided movement execution. One study investigated ROM during functional reach tasks in people with PD and found that ROM was significantly larger during the real world task compared to the fully-immersive VR task (Boucher et al., 2013). Regarding a grasping task with moving and stationary targets, Wang et al. (2011) found that success rates were lower and movement time longer in the VR condition, when compared to a non-immersive VR setup. However, these studies focused mainly on task paradigm, not providing repetitive exercise to investigate movement execution.

Therefore, the aim of this study was to investigate the impact of VR on movement execution. In specific, we compared movement execution during fully immersive VR exercises with comparable exercises instructed by a therapist. The exercise set was designed to improve ROM and postural control and incorporated elements derived from the Lee Silverman Voice Treatment (LSVT) BIG therapy, Tai Chi, and balance training, all of which have demonstrated efficacy in improving motor function, especially ROM and balance, in individuals with PD (Li et al., 2012; Ebersbach et al., 2010). Given that VR environments may pose greater demands on cognitive–motor integration, whereas therapist instructed (TI) exercises may allow for safer yet more intensive execution, we hypothesized that ROM would be greater and movement execution smoother during the therapist-instructed exercises.

## 2 Methods

This interventional study compared the immediate effect of VR on movement execution. With a cross-over design, participants performed VR-instructed exercised followed by TI exercises, or vice versa, within one session of exercise.

### 2.1 Participants

Potential participants were initially screened and recruited using a convenience sampling approach during their inpatient rehabilitation stay at Segeberger Kliniken. They were included in the study at least 2 weeks after their discharge. Inclusion criteria was a diagnosis of PD. Exclusion criteria were other neurological disorders and orthopedic or other conditions that affect the movement during the protocol or decline in cognition that could affect the understanding of the protocol (Mini Mental State Examination ≤24). Participants were examined ON medication. All assessments were carried out at the movement laboratory of the MSH Medical School Hamburg. This study was approved by the local ethics committee of the MSH Medical School Hamburg, and all participants gave written and informed consent prior to participation.

### 2.2 Virtual reality setup and exercises

We used the CUREO® 2 by CUREosity (Düsseldorf, Germany) for this study. It is a fully-immersive VR setup which consists of a head mounted display and two handheld controllers and is remotely controlled via a tablet by the therapist. The advantage of CUREO® is that this VR application was specifically designed for neurological patients, hence it is much more adjusted to the motor and cognitive abilities of people with PD. The system is used in clinical routine in various different (neuro-)rehabilitation settings and has been evaluated for people with PD (Heinzle et al., 2026). We chose three different VR exercises for this investigation, based on equivalent exercises used in physiotherapy to have a high relation to clinical practice. Two of the exercises (Torso Twist and Rising Hands) were mainly focused to increase ROM of the upper limbs and were performed sitting on a chair using the provided handheld controllers. People with PD generally show hypokinesia and bradykinesia, meaning movements are slow and have a small amplitude (Jankovic, 2008). Training movement amplitude was found beneficial for people with PD as was shown with concepts like LSVT-BIG (McDonnell et al., 2018). Furthermore, trunk mobility can be limited due to rigidity and even associated with quality of life (Cano-de-la-Cuerda et al., 2020), therefore increasing ROM of the trunk can be clinically relevant. Movement amplitude was calibrated in the application according to arm span of participants. The following two exercises were chosen with the aim to increase ROM:

*Torso Twist*: Participants were asked to hold a virtual ball in front of their chest with their arms stretched out and then move that ball along a given trajectory to each side by twisting their torso. The end of the trajectory was marked as well as an acoustic signal provided when they reached the end. Participants could see their virtual avatar and also see a mirrored version of their avatar in front of them for visual feedback. Furthermore, verbal feedback was given if participants diverted form the given trajectory with one or both hands. This exercise was repeated 5 times.

*Rising Hands*: This exercise was similar to the Torso Twist exercise as participants also had to follow a given trajectory with their hands. However, this time the trajectory was going up and down with both arms stretched out to the front as the starting point. Similar to the previous exercise the ends of the trajectory were marked visually, and the acoustic signal provided when participants met the target. Participants could again see their mirrored avatar and got verbal feedback if they diverted from the trajectory with one or both hands. This exercise was repeated 5 times.

**Figure 1:**
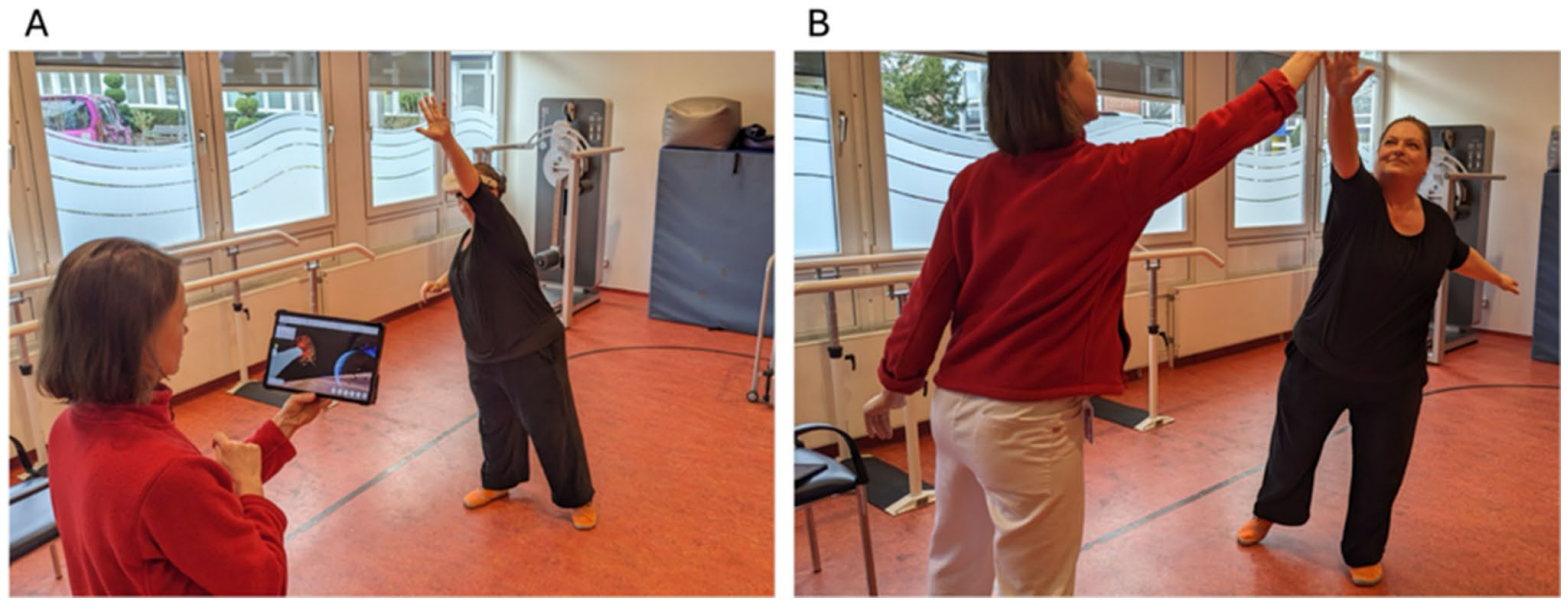
Meteor exercise with VR **(A)** and TI **(B)**.

The third exercise (Meteor, Figure 1) was performed while standing and focusing on postural control with an additional cognitive component. Postural instability is one of the key features of PD (Jankovic, 2008) and can be treated through exercise. Several forms of exercise were found to be beneficial for improving postural control, including exergaming (Wang et al., 2023). Hence, the following exercise was chosen for the aim to train postural control. Movement amplitude was calibrated in the application according to the height of participants.

*Meteor*: This exercise had more of a gamified character. We used the hand tracking function where virtual hands are visualized in the VR environment based on the participants actual hands, which are tracked by exterior cameras of the head mounted display. Participants were standing in an outer-space-like environment and saw meteors in the form of handprints flying towards them. The task was to catch the handprints with their hands stretched out (like a high-five). Participants got acoustic feedback when they hit the targets correctly (Figure 1). The handprints were displayed in red and blue color, and they had to use the respective side of the virtual projection of their own hand, which was also shown in red and blue. Verbal feedback was provided by the software if participants used the incorrect hand or did not open their hand sufficiently. For this task participants needed to shift their body weight to reach the handprints and have appropriate reaction times, hence more cognitive resources were needed compared to the other two exercises. This exercise lasted 60 s. As this exercise was performed during standing while wearing the mead-mounted display participants were secured with a safety harness.

Equivalent TI exercises were conducted using soft balls for the Torso Twist and therapy rings for the Rising Hands. Standardized instructions were given by the therapist as well as a demonstration. During the exercise the therapist carried on with the demonstration as well to mirror the participants movement and increase comparability with the VR exercise. For the Meteor exercise we used stickers on the back of the hand as well as soft balls which participants had to strike with their hands, to mimic the task in the VR environment. The TI exercises comprised the similar movements and were conducted with the same number of repetitions or for the same time, to ensure highly comparable intensity.

The order (VR or TI exercises first) of the exercises conditions was block-randomized while the order of the three exercises (Torso Twist, Rising Hands and Meteor) was kept the same across participants. The same number of participants performed the VR or the TI exercises first.

### 2.3 Assessment of movement

**Figure 2:**
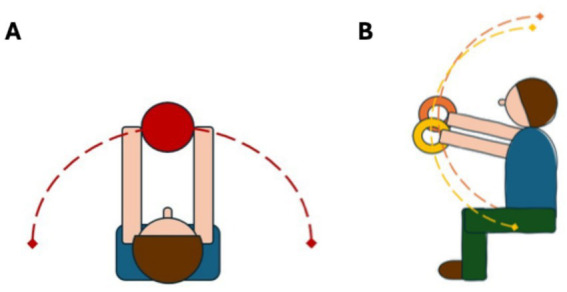
Movement trajectories of **(A)** Torso twist-like exercise from the top view; **(B)** Rising hands-like exercise from the side view; The dotted lines represent the trajectory of the ball or therapy rings with the end points, which are the turning points.

During both ROM exercises (Torso Twist and Rising Hands) movement was assessed via reflective markers (Qualisys, Sweden, 16 mm markers, sampling frequency: 85 Hz, 12 cameras) placed on the wrists (distal radial head). After calculating the mean between both points, we tracked its arc length which was defined as the total distance covered for each full twist or rise (from the maximum excursion of one side to the other/top down, Figure 2). This was done using a customized MATLAB script (MathWorks, USA). For the Meteor exercise we recorded the center of pressure (COP) via a pressure platform (Noraxon, myoPRESSURE, USA, sampling frequency: 300 Hz) which was integrated within a treadmill (HP-Cosmos, Germany), which participants were standing on. The variables computed were COP path length, COP velocity and COP ellipse area.

### 2.4 Clinical assessment

The following clinical tests were conducted to characterize the participants with regard to disease severity and cognition: Movement Disorder Society Unified Parkinson’s Disease Rating Scale (MDS-UPDRS) part III and Montreal Cognitive Assessment (MoCA).

### 2.5 Statistical analysis

Statistical analysis was conducted using R Studio software (R Development Core Team, 2010). Outcomes were checked for normality of distribution and log-transformed if necessary. For all outcomes (dependent variables) we calculated a one-way Anova, with repeated measures, with test condition (2 levels: VR vs. TI) and order (2 levels: VR first or TI first) as the independent variables and with case-wise deletion for missing data. *p*-values were Bonferroni-adjusted to correct for multiple testing. Additionally, Cohen’s d was calculated to estimate effect size. Effect sizes were interpreted as small (≥0.2), medium (≥0.5), or large (≥0.8) (Rosenthal, 1996).

## 3 Results

Sixty-two individuals with idiopathic PD were initially screened for this study. Reasons for participants not being able to participate were the inability to reach the study site, limited availability, or orthopedic problems. Finally, 30 participants fulfilled all in- and exclusion criteria and were included in this experiment. Participants had a mean age of 61.5 (SD: 9.2), a mean MDS-UPDRS III value of 19.4 (SD: 7.8) and a mean disease duration of 5.3 (SD: 2.7) years (Table 1).

**Table 1:** Participant characteristics (*n* = 30).

Variable	Mean (SD)
Age (yrs)	61.5 (9.2)
Height (cm)	177.7 (9.5)
Weight (kg)	84.6 (15.7)
Sex (m/f)	21/9
MDS-UPDRS-III	19.4 (7.8)
Disease duration (yrs)	5.3 (5.0)
MoCA	26.6 (2.7)

Values represent mean (SD); m, male; f, female; MDS-UPDRS-III, Movement Disorder Society-Unified Parkinson’s Disease Rating Scale Part III; MoCA, Montreal Cognitive Assessment.

**Figure 3:**
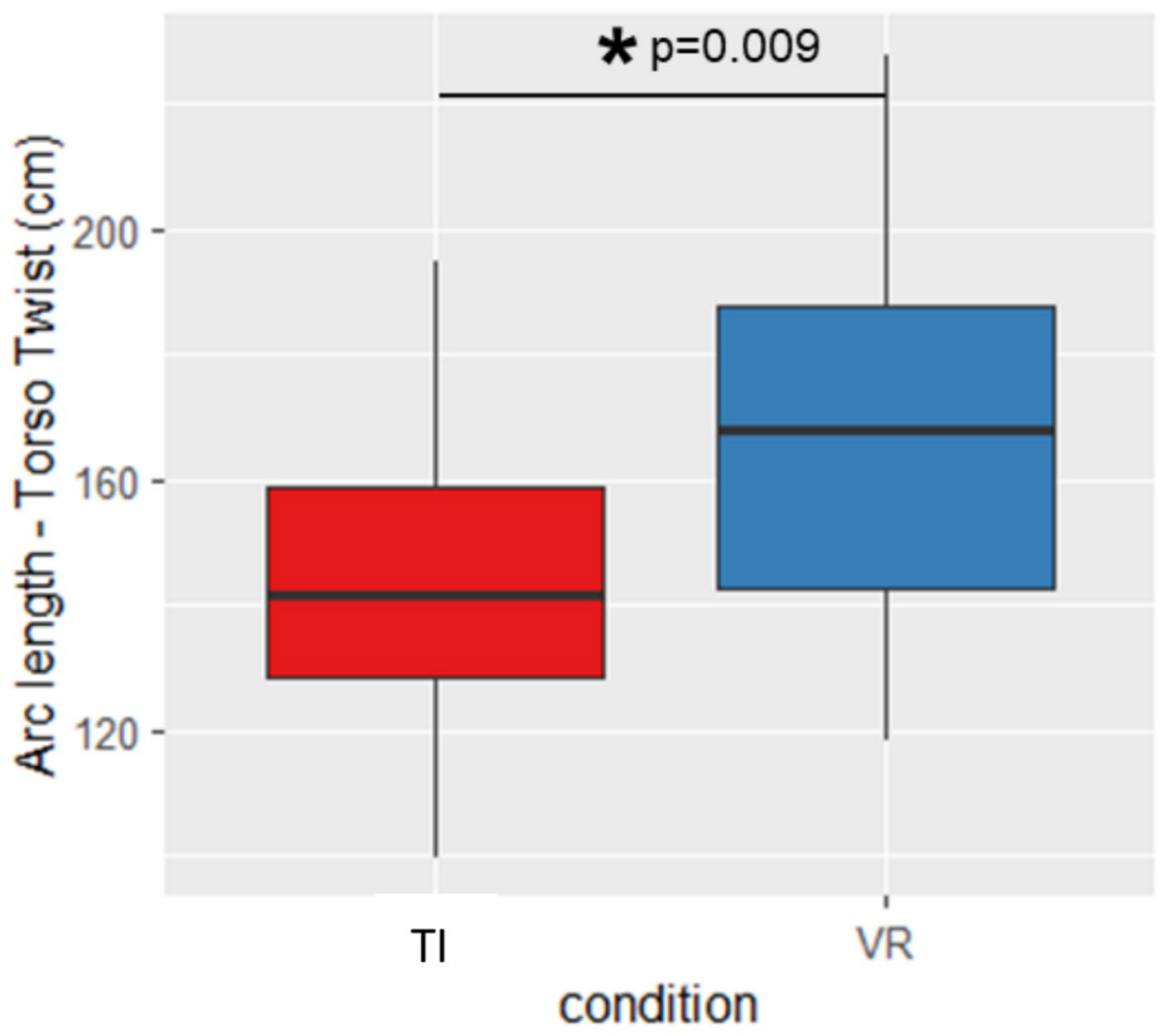
Arclength during torso twist. TI, therapist instructed, VR, virtual reality, *Significant different (*p* < 0.05); error bars indicate standard deviation.

**Figure 4:**
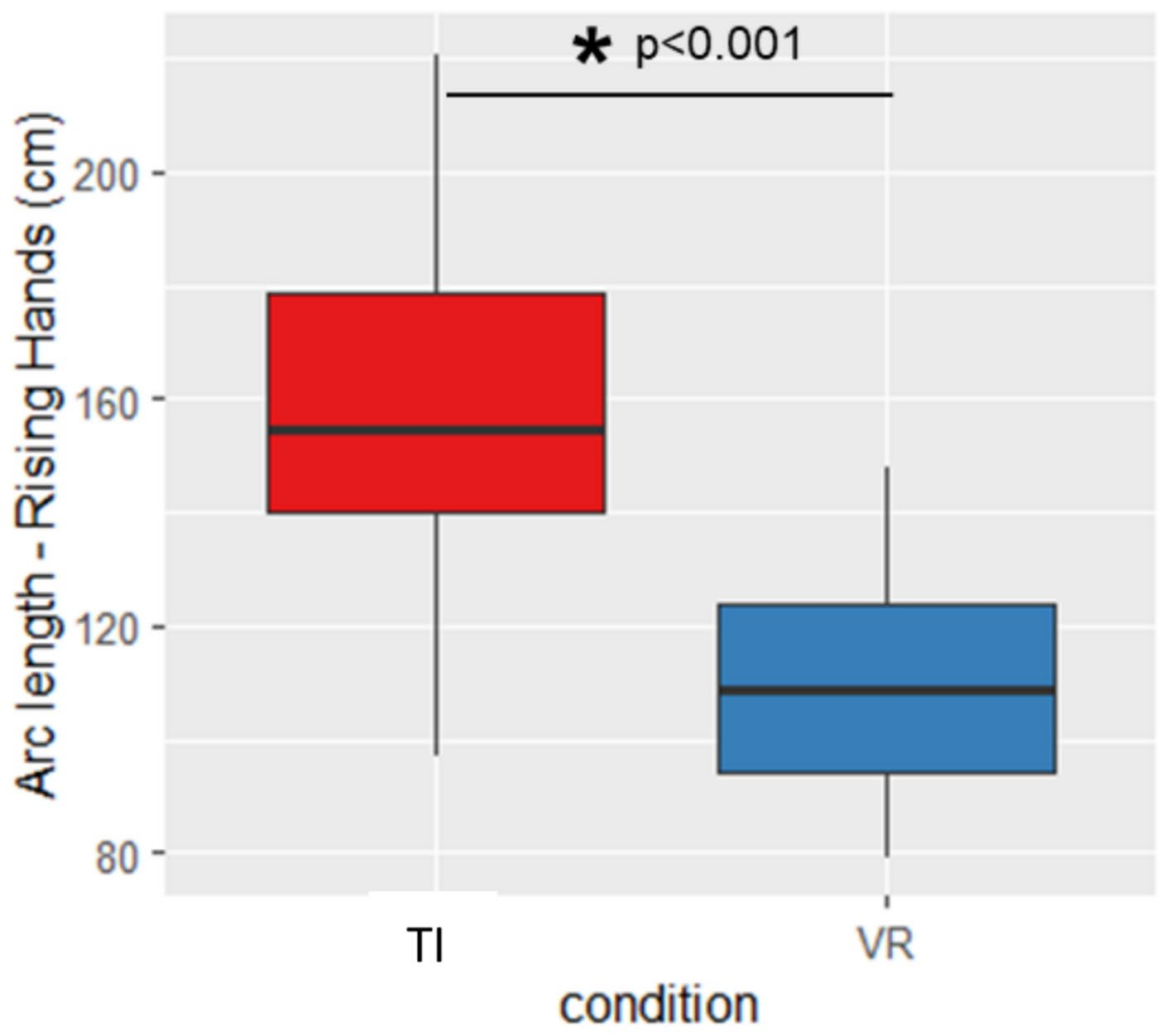
Arclength during rising hands. TI, therapist instructed, VR, virtual reality, *Significant different (*p* < 0.05), error bars indicate standard deviation.

For Torso Twist we found that arclength was significantly greater indicating larger ROM during the VR exercise compared to the TI condition (*p* = 0.009, d = 0.895) (Figure 3). Conversely, during Rising Hands arclength was significantly larger for the TI condition than for the VR exercise (*p* < 0.001, d = 1.955, Figure 4).

For the Meteor exercise and the Meteor comparable exercises, we found that participants moved their COP significantly faster (*p* < 0.001, d = 1.057), covered a longer path length (*p* < 0.001, d = 1.077) and also covered a larger COP ellipse area (*p* = 0.024, d = 0.599) during the TI condition compared to the VR condition. For details see Table 2.

**Table 2:** Results of the Meteor and Meteor-comparable physical therapist instructed exercise.

Variable	Exercise condition	Mean (SD)	*p*-value
COP path length (cm)	TI	1553.84 (775.30)	<0.001*
	VR	860.84 (475.61)	
COP velocity (cm/s)	TI	25.90 (12.92)	<0.001*
	VR	14.52 (8.05)	
COP ellipse area (cm^2^)	TI	409.09 (345.93)	0.024*
	VR	232.52 (231.91)	

COP, center of pressure; TI, therapist instructed condition, VR, virtual reality condition, SD, standard deviation; * significantly different after Bonferroni correction for multiple comparisons.

For none of the exercises there was any effect of order on the outcome of interest. Furthermore, there was no significant interaction effect between condition and order.

## 4 Discussion

This study investigates the impact of VR on exercise execution in comparison to conventional TI exercises. For the exercises with the aim to increase ROM, inconsistent results were found. While a greater ROM was observed for the Torso Twist exercise in the fully immersive VR condition, the Rising Hands exercise resulted in a higher ROM in the TI condition. Those divergent findings may be partially explained by the direction of vision and the associated availability of visual feedback. During the Torso Twist exercise, participants direct their gaze laterally while rotating the torso. In this context, the VR system continuously provides visual feedback throughout the entire movement. In contrast, in the TI condition, participants lose visual contact with the therapist toward the end of the lateral rotation, thereby receiving less visual feedback at these critical points of the movement. The increased amount of continuous visual feedback and the gamified component of reaching a target in the VR condition may have enhanced exercise intensity and contributed to the higher ROM values observed compared to the TI condition. This interpretation is supported by another study in which the authors found altered movement execution during augmented reality compared to real world due to altered delivery of visual information in PD (Boucher et al., 2013). By contrast, in the Rising Hand exercise—during which constant feedback was provided in both the TI and VR conditions—participants may perform better in the TI condition, as the feedback is more individualized, being delivered by a therapist who can adapt it to each participant’s performance in real time.

For the exercise aimed at enhancing postural control by improving limits of stability we found a larger COP displacement and faster COP excursion during the TI condition compared to the VR condition with large effect sizes between the groups. Greater and faster COP excursions are indicative of higher exercise intensity during balance tasks and may reflect increased movement demands. One possible explanation is that participants felt safer in the presence of a therapist in the TI condition while also having unobstructed visual input, which may have encouraged them to explore their limits of stability more extensively than in the VR condition. Another potential explanation for the observed differences between TI and VR conditions is the limited individualization of the VR intervention, as suggested by the greater variability in the data observed in the TI condition. Nevertheless, these interpretations remain speculative, and further research is required to clarify the underlying mechanisms and to draw more definitive conclusions. Our results appear to be robust against potential carry over effects as the order of the VR and TI conditions were randomized, and we did not find any significant effect of order or any order by condition interaction.

Very limited literature exists investigating the VR system by CUREO® which we used in our study including an investigation of the effect of the VR system on movement execution in individuals with PD. Hence it is challenging to put our results into perspective to previous research. One study using the same device was conducted to improve upper limb motor function in people post stroke (Lülsdorff et al., 2023). The authors found VR to be similar effective compared to TI exercises after a training period of 3 weeks and conclude that VR can be a promising addition to conventional therapy (Lülsdorff et al., 2023). Our findings extend the current literature by showing that the effects of fully-immersive VR interventions on movement execution depend on the type of exercise.

We have shown that for some exercise types, training intensity provided by the VR system might not be optimal as indicated by better motor performance during the TI exercises. This may be improved in the future by a closer collaboration of technology-professionals, therapists and the targeted patient group as well as more personalized VR rehabilitation options based on the individual deficits (Canning et al., 2020). Some preliminary research has addressed this by investigating ways to improve VR for people with PD using over- and under-scaling of the movements within the virtual environment during a reach task to improve reach and deemed this an important factor (Shahnewaz Ferdous et al., 2022).

In terms of safety and feasibility we neither had incidences of nausea, dizziness nor falls experienced by participants during this trial, indicating the VR system we used is safe. With regard to feasibility, we had two instances where participants were not able to reach the targets for the Meteor exercise and no immediate options were available for therapists to adapt the exercise. In addition, some participants had trouble understanding the instructions provided for the VR exercises, hence the examiner had to intervene by giving additional explanations for the exercise. The difficulties in understanding the instructions given by the VR software supports the idea that the development of VR devices would benefit from input from patient groups and health professionals who are experienced with exercise therapy to improve usability of the devices. Furthermore, there were technical issues with the VR application which sometimes led to delays in the study protocol. The most common problems were delays due to unsuccessful pairing of the head mounted display with the tablet and problems with the calibration of the device, that led to incorrect depiction of the virtual elements in the VR environment. Those problems led to time delays, but were always resolved before starting the exercises.

### 4.1 Limitations

The following limitations can be acknowledged: First, the VR device which was used was not validated scientifically before, so we do not have information about the accuracy or potential delay of movements in the VR environment. Second, given the cross-sectional design of this study and hence the lack of a prolonged training period, we did not investigate usability and acceptability of the VR application in the long-term. Furthermore, regarding the comparability of the two exercise conditions in this study some exercises were not standardizable to the full extent as the speed of presenting the soft balls during the real-world Meteor game was controlled by the therapist. Additionally, we did not record subjectively rated intensity or difficulty level for the exercises. This should be implemented in future studies to ensure adequate levels of intensity. Lastly, our results cannot directly be transferred to other available VR systems.

## 5 Conclusion

This study demonstrates that the effects of VR on exercise execution differ depending on the specific exercise goal and task characteristics. While fully immersive VR was associated with greater ROM in exercises that benefit from continuous visual feedback, such as the Torso Twist, conventional therapy-instructed exercises resulted in superior performance for other tasks, including the Rising Hands exercise and balance exercises targeting limits of stability.

The findings suggest that visual feedback, perceived safety, and task-specific demands play a crucial role in determining the effectiveness of VR-based interventions. In particular, the presence of a therapist may facilitate higher exercise intensity during balance tasks, whereas immersive VR may enhance movement execution in exercises requiring sustained visual guidance.

Overall, VR cannot be considered a universal substitute for conventional therapy but rather a complementary tool whose effectiveness depends on exercise design and individualization. Future research should focus on optimizing VR interventions through adaptive feedback and personalized task difficulty to fully exploit their potential in therapeutic exercise settings.

## Data Availability

The datasets presented in this article are not readily available because the raw data supporting the conclusions of this article will be made available by the authors, if ethical restrictions allow. Requests to access the datasets should be directed to Christian Schlenstedt, christian.schlenstedt@medicalschool-hamburg.de.
